# Supplementary material for: The deletion of HK-1 gene affects the bacterial virulence of Pseudomonas stutzeri LH-42
Source: PLoS One. 2022 Nov 29;17(11):e0277089. doi: 10.1371/journal.pone.0277089 (PMC9707753; doi:10.1371/journal.pone.0277089)
Supplement: S1 Raw image — (PDF) [file pone.0277089.s001.pdf]

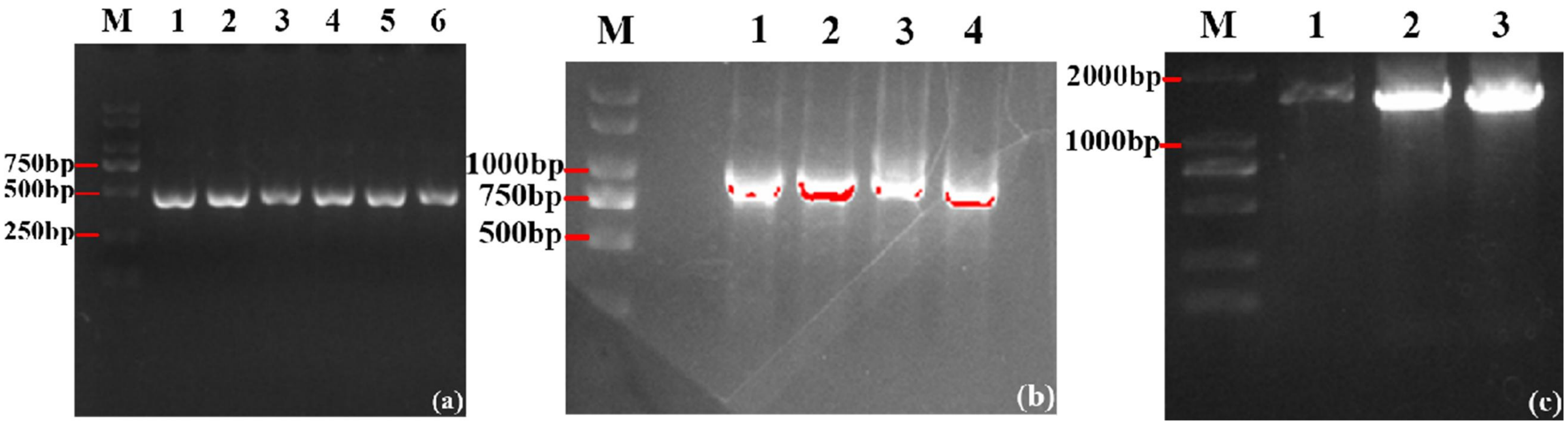

Figure (a) Result of PCR amplification of left and right homology arms.
(b) Result of Km resistance gene PCR amplification gel electrophoresis.
(c) Results of homologous fragment gel electrophoresis.
